# Integrated morpho-physiological and transcriptomic dissection of waterlogging tolerance in *Hibiscus hamabo*

**DOI:** 10.3389/fpls.2026.1882944

**Published:** 2026-09-11

**Authors:** Huizi Liu, Sheng Yang, Zhixia Zhao, Xuan Wang, Xiaomin Zhang, Dandan Kong, Siyang Jin, Qiuxia Chen, Xing Liu

**Affiliations:** 1 Zhejiang Institute of Subtropical Crops, Zhejiang Academy of Agricultural Sciences, Wenzhou, China; 2 Zhejiang Forestry Resources Development Co., Ltd., Wenzhou, China; 3 Engineering Research Center for Southeast Coastal Characteristic Plants of National Forestry and Grassland Administration, Wenzhou, China

**Keywords:** *Hibiscus hamabo*, morpho-physiology, transcriptomic analysis, waterlogging tolerance, weighted gene co-expression network

## Abstract

*Hibiscus hamabo* is an important coastal ornamental plant and a pioneer tree species for coastal protection forests. Prolonged waterlogging severely restricts its ecological expansion toward the intertidal‑zone forefront and limits population regeneration; clarifying its waterlogging‑adaptation mechanisms is essential for coastal wetland ecological restoration. In this study, phenotypic, physiological and transcriptomic responses of *H. hamabo* under 3-, 9-, and 18-day waterlogging treatments were characterized. Prolonged waterlogging markedly suppressed plant growth and biomass accumulation, and impaired photosynthetic performance via pigment degradation and stomatal closure. To counteract oxidative injury, *H. hamabo* sequentially triggered osmotic‑adjustment and antioxidant‑defense systems, relying on soluble sugars, proline and ROS‑scavenging enzymes. Transcriptome profiling identified stage‑dependent gene expression changes mainly related to photosynthesis, hormone signaling and phenylpropanoid biosynthesis. WGCNA uncovered two core co‑expression modules with opposite functional roles, representing stress‑defense activation and growth repression, respectively, and hub genes for osmotic homeostasis, ROS detoxification and photosynthesis preservation were screened. Collectively, our findings illustrate the coordinated adaptive strategy of *H. hamabo* against waterlogging stress and supply promising candidate genes for breeding waterlogging‑tolerant coastal woody plants.

## Introduction

As sessile organisms, plants have evolved adaptive mechanisms to modulate morphology, physiological, biochemical, and metabolic changes, thereby enhancing their survival under hypoxia and enabling them to endure prolonged submergence stress (Jia et al., 2021; Tang et al., 2021; Li et al., 2025; Wang et al., 2025). Morphologically, plants may exhibit growth inhibition, formation of aerenchyma and adventitious roots, thin gas films, enlarged lenticels, elongated internodes and petioles, and other adaptive strategies to alleviate hypoxic conditions (Hattori et al., 2009; Shimamura et al., 2010; Kuroha et al., 2018; Kurokawa et al., 2018; Loreti and Perata, 2020; Zhou et al., 2020; Koramutla et al., 2022; Yang et al., 2023; Li et al., 2025). For instance, to minimize oxygen loss, plants develop a radial oxygen loss (ROL) barrier by lignifying the outer cell walls of their roots (Voesenek and Bailey‐Serres, 2015). The ROL barrier in roots enhances the function of aerenchyma, promotes the longitudinal diffusion of internal root oxygen toward root tips, and thereby facilitates root growth in waterlogged soil (Helen et al., 2010; Ranathunge et al., 2011; Abiko et al., 2012; Pedersen et al., 2021; Shiono et al., 2022). As submergence stress persists, the plant base differentiates numerous adventitious roots that rapidly replace the hypoxia-damaged, functionally declining primary rootary essential survival strategy for efficiently capturing dissolved oxygen in flooded environments (Sauter, 2013). Concurrently, submergence triggers stomatal closure, impeding water exchange and gas uptake; the leaf gas film (LGF) then enhances underwater gas exchange, sustaining photosynthesis and cellular oxygen acquisition (Kurokawa et al., 2018). In addition, hypoxia typically promotes pronounced elongation of internodes and petioles while narrowing the stemowing angle, enabling the shoot to reach the water surface and mitigate hypoxic injury (Hattori et al., 2009; Li et al., 2025).

Physiologically, adjustments in photosynthetic efficiency, activation of antioxidant systems, and accumulation of osmotic regulators are common adaptive strategies to cope with energy crisis and oxidative damage caused by hypoxia (Sachdev et al., 2021; Liang et al., 2025; Meng et al., 2025). Photosynthesis is one of the most hypoxia-sensitive physiological processes, as it requires substantial energy and is tightly linked to chloroplast structure and function (Fukao et al., 2019). Flood-induced reductions in light availability, increased ethanol production, decreased intercellular CO_2_ concentration, stomatal closure, and chlorophyll degradation often lead to decreased photosynthetic rates, limiting carbohydrate accumulation and plant growth (Tamang et al., 2014; Sasidharan and Voesenek, 2015; Jia et al., 2021; Wang et al., 2025). For instance, in rice (*Oryza sativa*), submergence stress leads to the inhibition of chlorophyll degradation and the delay of leaf senescence (Ismail et al., 2013). Similarly, waterlogging in soybean (*Glycine max*) results in stomatal closure and reduced CO2 assimilation, which exacerbates growth inhibition (Tamang et al., 2014).

Hypoxia-induced anaerobic respiration not only reduces energy production efficiency but also leads to the overaccumulation of reactive oxygen species (ROS), such as superoxide anion (O_2_^-^), hydrogen peroxide (H_2_O_2_), and hydroxyl radicals (OH^-^) (Hasanuzzaman et al., 2020). These ROS can cause lipid peroxidation, protein oxidation, and nucleic acid damage, ultimately disrupting cell membrane integrity and normal metabolic processes (Wang et al., 2025). To counteract ROS toxicity, plants have evolved sophisticated antioxidant defense systems, including enzymatic components such as superoxide dismutase (SOD), peroxidase (POD), catalase (CAT), ascorbate peroxidase (APX), glutathione S-transferase (GST), glutathione per-oxidase (GPX), monodehydroascorbate reductase (MDHAR), and dehydroascorbate reductase (DHAR), as well as non-enzymatic antioxidants like soluble sugars (SS), soluble proteins (SP), ascorbic acid (AsA), glutathione (GSH), carotenoids, αarotenoids, and free proline (Pro) (Hasanuzzaman et al., 2020; Sachdev et al., 2021; Yuan et al., 2023). Malondialdehyde (MDA) arises as a terminal product of lipid peroxidation, generated when reactive oxygen species attack macromolecular targetsleculareDA the polyunsaturated fatty-acid side chains of phospholipids, enzymes, membrane receptors, and nucleic acidsicrs,id biological membranes (Han et al., 2017; Lu et al., 2023). The activation of these systems is crucial for maintaining redox homeostasis and enhancing flood tolerance. For example, in ramie (*Boehmeria nivea*), waterlogging-tolerant varieties exhibit higher SOD and POD activities and lower malondialdehyde (MDA, a marker of lipid peroxidation) content compared to sensitive varieties (Shao et al., 2024). The maintenance of ROS homeostasis during rice recovery from submergence stress can be attributed to the elevated levels of ascorbic acid and glutathione reductase activity, which ensure optimal functioning of the ascorbic acid-glutathione cycle and enhance the efficiency of H_2_O_2_ detoxification (Ella et al., 2003).

At the molecular level, plants respond to hypoxia through complex transcriptional regulatory networks. Central to this process is the oxygen-dependent N-end rule pathway, which orchestrates the turnover of the ethylene response factor VII (ERF-VII) family proteins (Gibbs et al., 2011; Licausi et al., 2011; Guo et al., 2024). Moreover, mitogen-activated protein kinases MPK3/6 and calcium-dependent protein kinase CPK12 play pivotal roles in hypoxic signal transduction by phosphorylating ERF-VII proteins (Zhou et al., 2022; Fan et al., 2023; Guo et al., 2024). Transcriptional regulation of key genes in hypoxia signaling is also critical (Guo et al., 2024). In *Arabidopsis thaliana*, natural variation in the cis-elements of the *RAP2.12* promoter that are bound by WRKY70 has been linked to elevated *RAP2.12* expression, implying an adaptive response to humidity (Lou et al., 2022). In addition, WRKY33 and WRKY12 have been shown to directly regulate *RAP2.2* (Liu et al., 2021; Tang et al., 2021). In sorghum, the expression of the miR528-encoding gene is directly activated by WRKY76, which in turn targets the superoxide dismutase 2 (SOD2) gene to modulate reactive oxygen species (ROS) scavenging. Overexpression of WRKY76 markedly reduces submergence tolerance, demonstrating that the WRKY76trating module acts as a negative regulator in sorghumor response to flooding stress (Meng et al., 2025). Transcriptomic studies in various plant species, including *Arabidopsis thaliana*, rice, and lotus (*Nelumbo nucifera*), have revealed stage-specific expression patterns of DEGs during flooding stress (Deng et al., 2022). Early responses (within 3 days) often involve genes related to energy metabolism and hormone signaling, while long-term responses (10 days or more) prioritize cell wall modification, secondary metabolism, and ROS scavenging pathways (Deng et al., 2022). For example, in lotus, DEGs enriched in photosynthesis and ethylene signaling pathways are activated in the early stages of submergence, whereas genes involved in secondary metabolism and pathogen defense dominate in later stages (Deng et al., 2022).

Global climate change-driven sea-level rise, anomalous tides, and recurrent coastal flooding increasingly constrain the survival and ecological functional stability of littoral vegetation (Macreadie et al., 2021). With a long coastline and abundant coastal wetland resources, China harbors wetland ecosystems that integrate terrestrial carbon sequestration and marine biogeochemical cycling, representing some of the most productive and ecologically functional ecosystems on Earth (Macreadie et al., 2021). It is also a globally significant “blue carbon” reservoir. Its carbon sequestration capacity far exceeds that of terrestrial ecosystems such as forests and grasslands, playing an irreplaceable role in mitigating global climate change and achieving the “dual carbon” goals (Anderson et al., 2022; Saintilan et al., 2023). Meanwhile, as a natural ecological barrier in the coastal zone, coastal wetlands perform key ecological service functions in resisting storm surges, mitigating wave erosion, purifying the environment, maintaining shoreline stability, providing habitats for rare and endangered species, and sustaining biodiversity (Li et al., 2022; Fluet-Chouinard et al., 2023). Faced with the increasingly severe risks of coastal flooding, there is an urgent need to expand and cultivate suitable tree species with strong stress resistance to ensure the health and stability of the coastal ecosystem.

*Hibiscus hamabo* Siebold & Zuccarini (*H. hamabo*), a member of the mallow family Malvaceae, is a deciduous shrub native to Asia and mainly distributed in coastal areas of Zhejiang Province, Fujian Province and other regions in China, as well as in Korea, Japan, and some Pacific islands (Li et al., 2012; Yan et al., 2014; Sakhanokho et al., 2020; Macreadie et al., 2021). *Hibiscus hamabo*, an endangered and highly attractive ornamental woody species, is also a pioneer tree species for coastal shelterbelt forests (Sakhanokho et al., 2020; Ni et al., 2022; Wang et al., 2022a; Liu et al., 2023a). *H. hamabo* is a semi-mangrove species that adapts to dramatic environmental fluctuations across the land-sea ecotone, featuring moderate waterlogging tolerance and capable of establishing stable communities in middle and low tidal zones (Wu et al., 2013; Wang et al., 2019; Ni et al., 2021, 2022; Liu et al., 2023a). It also possesses ecological potential for expansion to higher tidal levels, thus playing an irreplaceable role in enriching coastal woody plant resources and optimizing wetland community structure. As a natural coastal barrier, *Hibiscus hamabo* plays a positive role in resisting storm surges, mitigating wave erosion, purifying the environment and maintaining coastline stability (Wang et al., 2022a, 2022). With the completion of the *H. hamabo* genome assembly, a solid foundation has been established for systematic research on its waterlogging tolerance mechanism (Wang et al., 2022c). Therefore, investigating and dissecting the molecular mechanisms underlying *Hibiscus hamabo*’s response to submergence and its adaptive evolution under stress will provide a pivotal theoretical foundation and scientific basis for the genetic improvement of stress tolerance in coastal plants.

This study deciphers the morpho-physiological and molecular choreography of waterlogging tolerance in the coastal keystone species *H. hamabo*. Plants were subjected to 3, 9 or 18 days of submergence; phenotyping, photosynthetic trait analysis, biochemical assays and RNA-seq were integrated to chart dynamic responses. Progressive growth inhibition, pigment loss and photosynthetic decline were accompanied by phased activation of antioxidant and osmotic adjustment systems that curtail oxidative damage. Stage-specific transcriptomes revealed convergent regulation of photosynthesis, hormone signaling, biosynthesis of secondary metabolites, phenylpropanoid biosynthesis and MAPK signaling pathway. We further explored the co-expression patterns of stress-responsive genes and identified key regulatory networks associated with waterlogging adaptation. Critical genes involved in stress defense and growth regulation were characterized to reveal the core molecular mechanisms underlying stress tolerance. Collectively, the results provide a systems module for waterlogging adaptation in coastal woody plants and deliver gene resources for climate-resilient restoration and breeding of waterlogging stress shoreline vegetation.

## Materials and methods

### Plant materials and growth conditions

One-year-old *H. hamabo* seedlings with uniform size and growth vigor were used as experimental materials. The seedlings were sourced from Ninghai, Zhejiang Province, China. The experiment al operations and preservation of living plant stocks were conducted at the Germplasm Resource Garden of the Zhejiang Institute of Subtropical Plants in Wenzhou, Zhejiang. This region is characterized by a subtropical monsoon climate, with a mean annual temperature of 18 °C and an average annual precipitation of 1540 mm. No herbarium voucher specimens were prepared for this material, and plant materials are available from the corresponding author upon reasonable request.

### Waterlogging stress treatment

One-year-old *H. hamabo* seedlings with vigorous and uniform growth were selected for the waterlogging stress test, at least 20 plants per biological replicate, and a total of 3 biological replicates for waterlogging stress. The plants were divided into a waterlogging treatment group (WL) and a control group (CK). The WL seedlings were cultivated in rectangular tanks (520×355×285 mm) and subjected to persistent waterlogging conditions, with water supply replenished regularly to maintain stable flooding status throughout the experiment. The CK seedlings were grown under regular well-watered conditions beside the treatment tanks. Mature leaves from the fifth node were harvested at 0, 3, 9, and 18 d of treatment. All collected samples were immediately frozen in liquid nitrogen and stored at -80 °C for subsequent physiological, biochemical, and transcriptomic analyses.

### Scanning electron micrograph analysis

Scanning electron micrograph analysis was performed on one-year-old *H. hamabo*, with three biological replicates each for the CK and WL plants. The leaves from the fifth node were harvested and immersed in an electron microscopy fixative, fixed at room temperature for 2 h, and transferred to 4 °C for preservation. After dehydration and critical point drying, the specimens were sputter-coated with gold for 30 s. The samples were imaged using a SU8100 scanning electron microscope.

### Plant growth and the exchange of parameter measurement

Plant height and stem basal diameter of CK and WL plants were measured at 0, 3, 9, and 18d of waterlogging stress. Fresh weight was determined after blotting the plant surface dry with paper towels. Subsequently, the plant tissues were oven-dried at 60 °C until a constant weight was reached to obtain the dry weight.

Net photosynthetic rate (Pn) and stomatal conductance (Gs) of functional leaves were measured between 9:30 and 11:00 AM using a LI-6400 portable photosynthesis system (LI-COR, USA). For each measurement, uniform mature leaves located at the mid-section of branches with consistent light exposure were selected. Five readings were recorded per replicate and averaged. All measurements were performed with at least three biological replicates, and the resulting data were utilized for subsequent graphical and statistical analyses.

### Pigments, osmolytes, and antioxidant systems detection

Photosynthetic pigments (total chlorophyll, chlorophyll a, chlorophyll b, and carotenoids) were extracted from mature fifth-node leaves with 80% acetone and quantified at 470, 645, and 663 nm (Lichtenthaler and Wellburn, 1983). Biochemical indicators were determined using commercial kits (Suzhou Mengxi Biomedical Technology Co., Ltd., Suzhou, China). Following the manufacturer’s instructions, fresh leaf tissues were thoroughly homogenized in extraction buffer. The homogenate was centrifuged, and the resulting supernatant was used for reaction and absorbance detection. The following kits and methods were evaluated: soluble protein (SP) content by the Coomassie Brilliant Blue G-250 method (Cat. No. M1805A); soluble sugar (SS) content by the anthrone-sulfuric acid method (Cat. No. M1503A); proline (Pro) content by the ninhydrin colorimetric method (Cat. No. M0108A); superoxide dismutase (SOD) activity by the WST-8 method (Cat. No. M0102A); peroxidase (POD) activity by the guaiacol method (Cat. No. M0105A); catalase (CAT) activity by the ultraviolet absorption method (Cat. No. M0103A; Aebi, 1984); ascorbate peroxidase (APX) activity by monitoring ascorbate oxidation at 290 nm (Cat. No. M0403A); and malondialdehyde (MDA) content by the thiobarbituric acid method (Cat. No. M0106A). Each biochemical assay was performed in at least three biological replicates to ensure data reliability.

### Transcriptome sequencing and analysis under waterlogging treatment

Total RNA was isolated from the leaves of CK and WL plants using the Qiagen RNeasy Mini Kit (Qiagen, Hilden, Germany) with three biological replicates per treatment. RNA-seq libraries were constructed utilizing the NEBNext Ultra II RNA Library Prep Kit (NEB, Ipswich, MA, USA). RNA sequencing was conducted to identify waterlogging-responsive genes according to the protocols described by (Lin et al., 2013, 2014). Paired-end reads with a mean length of 150 bp were generated on the Illumina HiSeq™ X Ten platform. After trimming adapter sequences and filtering low-quality reads, the resulting clean reads were aligned to the *H. hamabo* reference genome using (Langmead and Salzberg, 2012). Raw counts were quantified and normalized following an established analysis pipeline (Lin et al., 2013). Differentially expressed genes (DEGs) were identified using DESeq2 (Love et al., 2014) with a false discovery rate (FDR) < 0.05, and no fold change (FC) cutoff was applied.

### Weighted gene co-expression network analysis

Weighted Gene Co-expression Network Analysis (WGCNA) was performed following the methodology described by (Langfelder and Horvath, 2008). This approach facilitates the systematic identification of gene expression patterns across multiple samples. By adhering to the principles of a scale-free network, the correlation coefficients of the expression matrix underwent power transformation (weighting). This process ensures that genes exhibiting high co-expression synergy are partitioned into the same functional clusters (modules) within the global network, ultimately categorizing the entire transcriptome into distinct co-expression modules.

### Statistical analysis

All experimental data were subjected to statistical analysis using SPSS (v.19.0, IBM, USA). For comparisons between two groups, the significance of differences was determined using two-tailed Student’s *t*-tests, with significance thresholds set at *P* < 0.05 (*) and *P* < 0.01 (**). To evaluate multiple groups, a one-way analysis of variance (ANOVA) was performed, followed by Tukey’s honestly significant difference (HSD) *post-hoc* test (*P* < 0.05). Detailed information regarding biological and technical replicates is specified in the respective figure legends.

## Results

### Effects of waterlogging stress on morphology in *H. hamabo*

**Figure 1:**
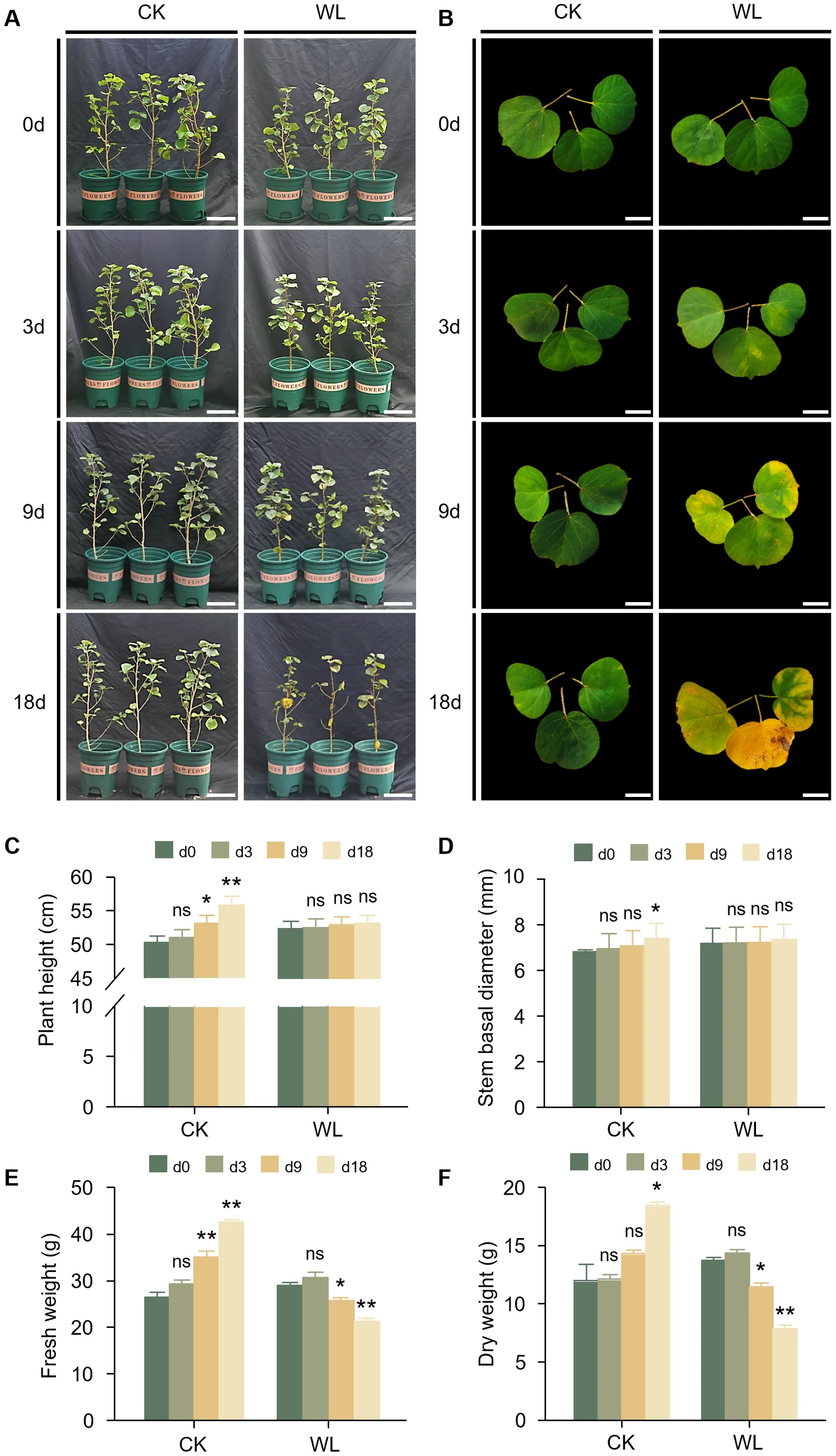
The effects of waterlogging stress on *H. hamabo* growth. **(A, B)** Phenotypes of the whole plant **(A)** and leaves **(B)** of one-year-old *H. hamabo* under waterlogging stress (WL) and non- waterlogging (CK) conditions. Bars in **(A)** and **(B)** represent 10 cm and 2 cm. **(C–F)** Statistical analysis of the plant heights **(C)**, stem basal diameter **(D)**, fresh weight **(E)**, and dry weight **(F)** for CK and WL plants. Error bars represent SE of 3 biological replicates, with a minimum of 10 plants per genotype in each replicate. Asterisks denote significant differences between 3-18d and 0d as determined by Student’s *t* test (*P < 0.05; **P < 0.01), while ns denotes no significant difference.

To investigate the effects of waterlogging stress on *H. hamabo*, we selected seedlings with uniform growth status from the same region for waterlogging treatment, and observed their phenotypic changes on the 3rd, 9th, and 18th days of treatment (3d, 9d, 18d), while using untreated plants as the control group (CK) for comparison. The results showed that the CK exhibited stable growth with spreading branches and dark green leaves throughout the 0~18 d period (**Figures 1A, B**). In contrast, the waterlogging stress group (WL) exhibited noticeable phenotypic abnormalities from 3d of stress exposure, characterized by leaf yellowing, wilting, and the development of brown leaf spots on some leaves. At 18d, leaf yellowing significantly intensified and was accompanied by extensive leaf shedding (**Figures 1A, B**). However, no significant differences in root systems were observed between the WL and CK groups during the waterlogging stress period (**Supplementary Figure 1**).

Statistical analysis indicated that the height of CK plants increased significantly by 5.50% and 10.93% on 9d and 18d of stress treatment compared to 0d, respectively (**Figure 1C**). A similar trend was observed in the change of plants diameter—by 18d, the diameter had increased significantly, rising from 6.81 mm to 7.37 mm (**Figure 1D**). Conversely, WL plants exhibited no significant changes in plant height and diameter throughout the stress treatment period. Additionally, CK plants demonstrated a significant effect on biomass accumulation. Compared with 0d, the fresh weight increased substantially by ~9 g at 9d, with a further significant increase of about 16 g at 18d (**Figure 1E**). The dry weight also showed a significant increase of approximately 6 g at 18d compared with 0d (**Figure 1F**). However, the biomass accumulation of WL was obviously inhibited. At 9d, fresh weight and dry weight decreased by 11.16% and 16.61% respectively, compared to day 0. By 18d these decreases were further exacerbated, reaching 26.47% and 42.53%, respectively (**Figures 1E, F**).

These results demonstrated that waterlogging stress has severely affected the growth and development of *H. hamabo*, leading to stunted plant height and diameter, as well as reduced biomass accumulation. Moreover, this inhibitory effect intensifies with the extension of stress time. These morphological deviations directly reflect growth suppression, suggesting potential functional impairment in the leaves, the primary photosynthetic organ. Such damage may diminish leaf morphology and root absorptive capacity, subsequently influencing photosynthetic pigment synthesis and the photosynthetic rate. Consequently, we next analyzed the photosynthetic characteristics of *H. hamabo* under waterlogging stress.

### Effects of waterlogging stress the photosynthetic system and stomatal morphology in *H. hamabo*

**Figure 2:**
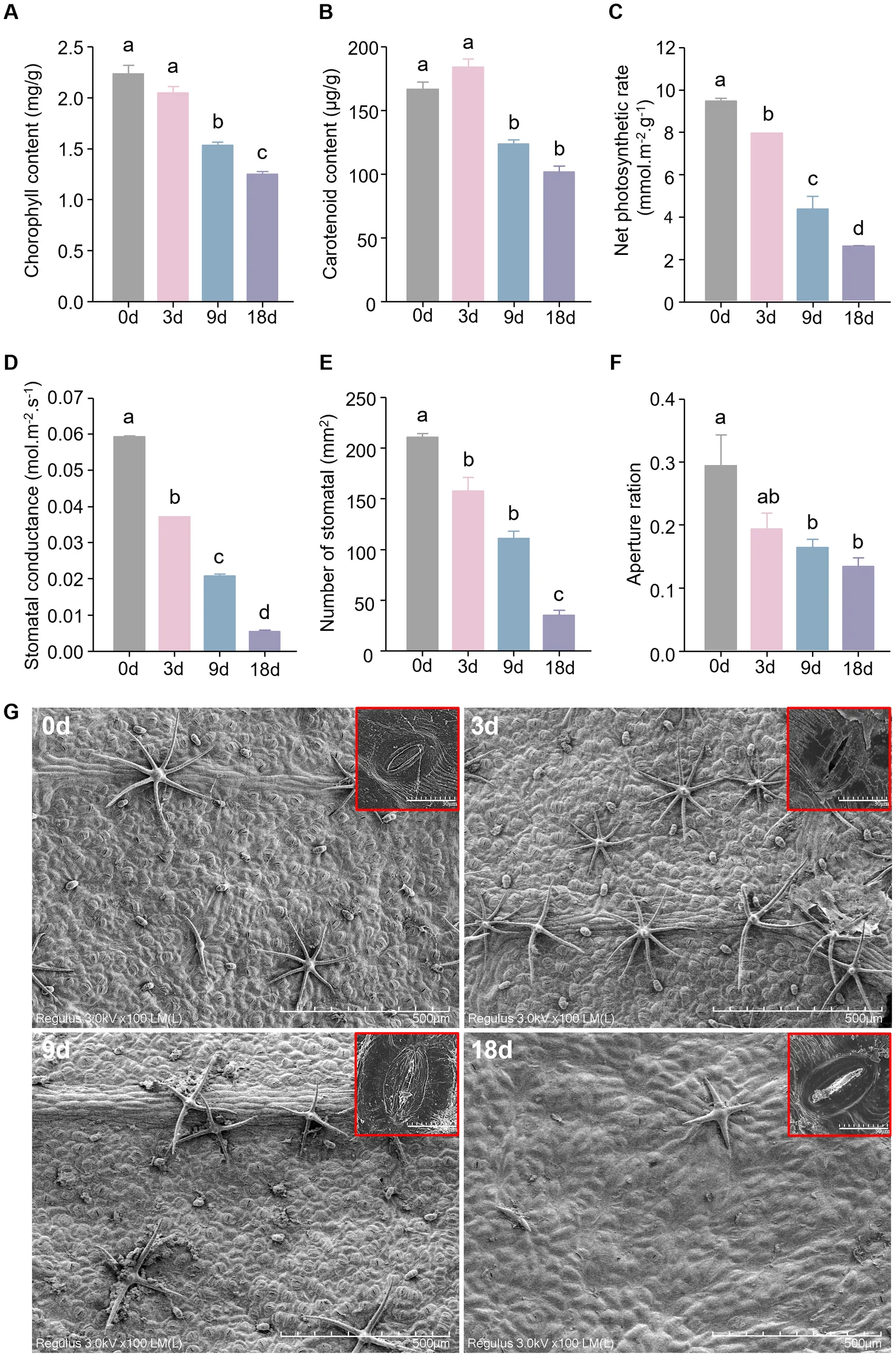
Waterlogging stress affects photosynthetic pigment content and stomata morphology traits in *H. hamabo*. **(A–F)** Statistical analysis of the chlorophyll content **(A)**, carotenoid content **(B)**, net photosynthetic rate **(C)**, stomatal conductance **(D)**, open stomata number **(E)**, and aperture ratio **(F)**. Error bars represent SE of 3 biological replicates, with a minimum of 3 plants in each replicate. Different letters indicate significant differences between 3d, 9d, 18d, and 0d (*P* < 0.05, one-way ANOVA, Tukey’s honestly significant difference (HSD) test). **(G)** Photographs of stomata on *H. hamabo* leaves on the 0th, 3rd, and 9th days of waterlogging stress treatment.

We tested the photosynthetic pigment content, net photosynthetic rate, and stomatal morphology of *H. hamabo* under different waterlogging durations. The findings revealed that, beginning at 3d, the chlorophyll content (**Figure 2A**), as well as the contents of chlorophyll a and chlorophyll b (**Supplementary Table 1**), decreased significantly. As the duration of stress increased, the decline in chlorophyllcontent became more pronounced, with the chlorophyll a/b ratio dropping from 2.168 at 0d to 1.264 at 18d (**Supplementary Table 1**). This is consistent with the degree of leaf yellowing in **Figure 1**. Furthermore, carotenoid content notably decreased at 9d of waterlogging stress, further dropping to 61.15% by 18d (**Figure 2B**). These results indicate that waterlogging stress weakens the light absorption capacity of leaves by disrupting pigment synthesis and the proportion balance.

The statistics of net photosynthetic rate (P_n_) and stomatal conductance (G_s_) showed that at 3d of waterlogging stress, P_n_ and G_s_ decreased by 16.08% and 37.44%, respectively, compared with 0d. At 9 days, the decrease expanded to 53.90% and 64.94%, and at 18 days, the P_n_ value dropped to 2.63 and the G_s_ value was almost 0 (**Figures 2C, D**). Meanwhile, waterlogging stress had a significant impact on the stomatal morphology of *H. hamabo* (**Figures 2E–G**). At 0d, the stomata exhibited greater openness with larger pore diameters. Subsequently, at 3d, the stomata partially closed, leading to a reduced aperture ratio of 0.19. By 9 and 18 days, the number of open stomata decreased to 111.14 and 35.15, respectively, with a corresponding decline in aperture ratio to 0.16 and 0.13 (**Figures 2E–G**). These changes may be the main cause of the decrease in P_n_ due to the lack of CO_2_ supply, which in turn worsens plant height and biomass reduction by reducing the accumulation of photosynthates.

### Effects of waterlogging stress on photosynthetic system and anaerobic respiratory system in *H. hamabo*

**Figure 3:**
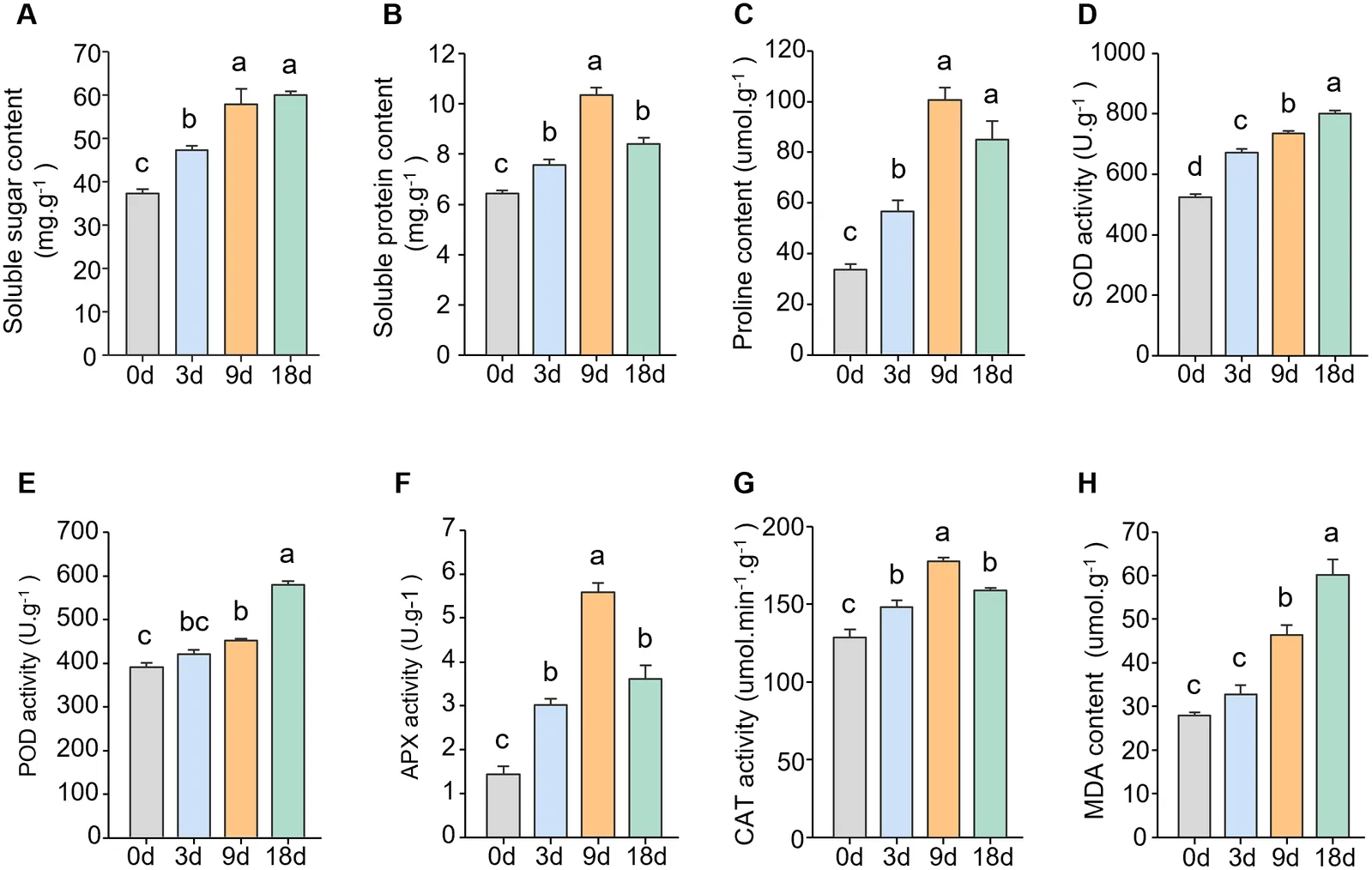
Physiological and biochemical responses of *H. hamabo* under waterlogging stress. Effects of waterlogging duration (0, 3, 9, and 18 d) on **(A)** soluble protein content, **(B)** soluble sugar content, **(C)** proline content, **(D)** superoxide dismutase (SOD) activity, **(E)** peroxidase (POD) activity, **(F)** ascorbate peroxidase (APX) activity, **(G)** catalase (CAT) activity, and **(H)** malondialdehyde (MDA) content under different waterlogging treatments. Error bars represent SE of 3 biological replicates. Different letters indicate significant differences between waterlogging stress treatment group and CK group (*P* < 0.05, one-way ANOVA, Tukey’s honestly significant difference (HSD) test).

To further investigate the effect of waterlogging stress on the physiological characteristics of *H. hamabo*, we measured the content of osmotic adjustment substances, antioxidant activities, and the content of MDA, an oxidative damage indicator, before and after exposure to stress. The results revealed that waterlogging stress significantly elevated the soluble sugar content in *H. hamabo*, with the increase rate correlating positively with the duration of stress, peaking maximum value of 59.99 mg/g at 18d (**Figure 3A**). The contents of soluble protein and proline reached their highest levels at 9 days, measuring 1.61 and 2.99 times greater than those at 0d, respectively (**Figures 3B, C**).

Under waterlogging stress, oxygen deficiency in plant roots causes disturbances in anaerobic respiration, leading to excessive generation of reactive oxygen species (ROS). To assess the ROS scavenging capacity of *H. hamabo*, we evaluated the activities of four key antioxidant enzymes—superoxide dismutase (SOD), peroxidase (POD), ascorbate peroxidase (APX), and catalase (CAT)—both before and after the stress treatment. We found that SOD and POD activities gradually increased with the extension of stress time and peaked at 18d, increasing by 53.39% and 47.71% respectively (**Figures 3D, E**). As the key enzymes for H_2_O_2_ in ROS scavenging system, APX and CAT activities increased progressively, peaking at 9d with increases of 289.48% and 37.90% over 0d, respectively (**Figures 3F, G**).

Despite the significant activation of the antioxidant enzyme system, the accumulation of reactive oxygen species (ROS) triggered by waterlogging stress still caused structural damage to cellular membranes, as evidenced by the dynamic changes in malondialdehyde (MDA) content. As a key indicator of membrane lipid peroxidation, MDA content increased significantly under stress. The increase was relatively gradual during the early stage (ta d), whereas the accumulation rate accelerated significantly between 9 and 18 d, reaching its peak at 18 d (60.12). These findings suggest that waterlogging exacerbates lipid peroxidation, with oxidative damage gradually increasing with prolonged stress (**Figure 3H**).

### RNA sequencing and identification of differentially expressed genes in *H. hamabo* under waterlogging stress

To evaluate the transcriptional regulatory responses of *H. hamabo* underwaterlogging stress, RNA-seq analysis was performed on leaf samples collected at 3 d, 9 d,and 18 d of treatment. The number of uniquely mapped reads for each library ranged from 20.48 to 30.18 million. All libraries exhibited high sequencing quality, with Q30 scores exceeding 98% and a stable guanine-cytosine (GC) content of approximately 44% (**Supplementary Table 2**), indicating the high reliability and quality of the RNA-seq data for downstream analysis.Furthermore, principal component analysis (PCA) revealed a clear separation among samples collected at different time points of waterlogging stress, suggesting distinct, stage-specific transcriptional signatures in response to the stress treatment (**Supplementary Figure 2**).

**Figure 4:**
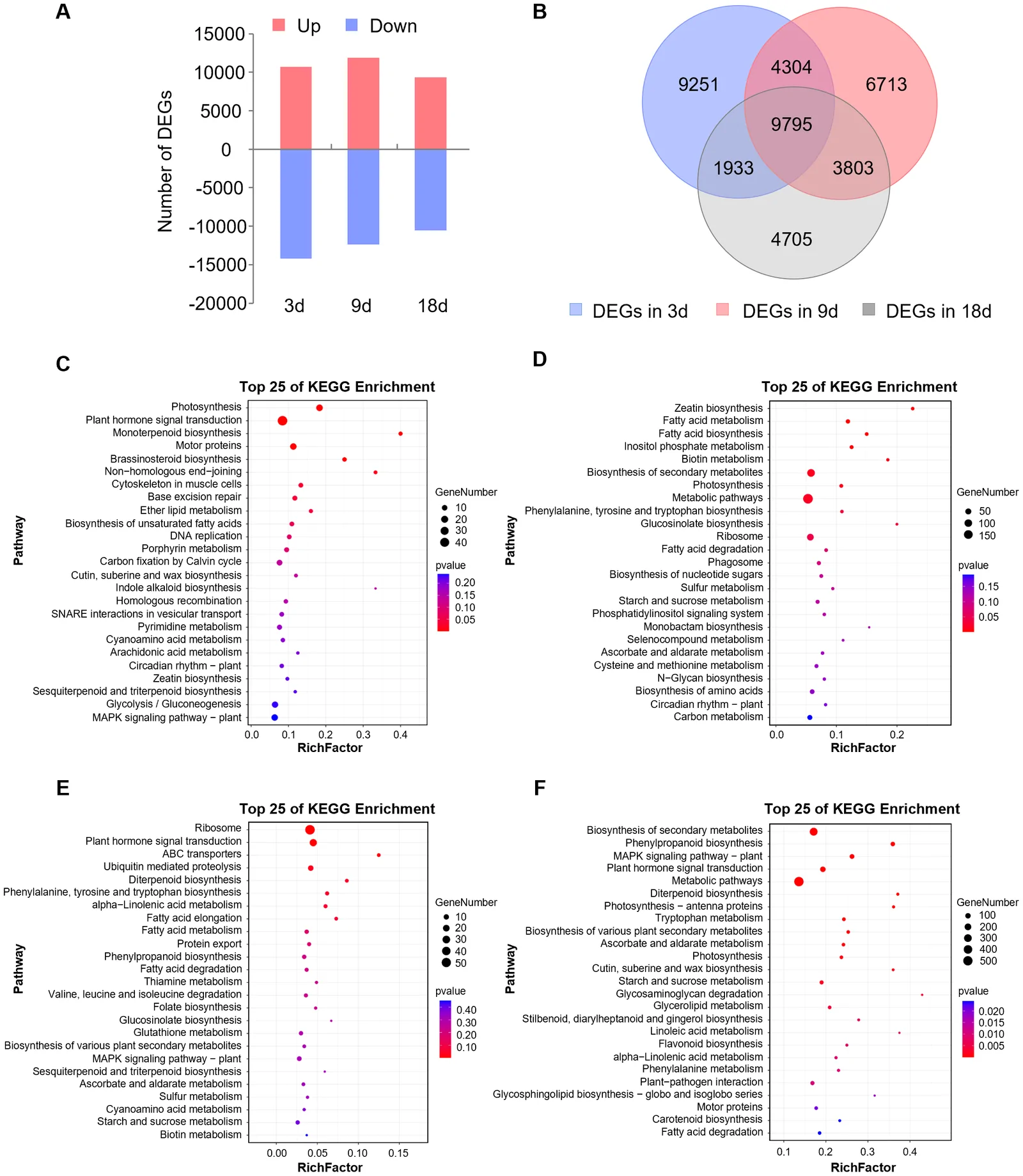
Transcriptomic characteristics and functional enrichment of differentially expressed genes (DEGs) in *H. hamabo* under waterlogging stress. **(A)** Number of up-regulated and down-regulated DEGs at 3, 9, and 18 d of waterlogging treatment compared to the 0d. **(B)** Venn diagram illustrating the overlaps and stage-specific DEGs among the three treatment durations. **(C–E)** KEGG pathway enrichment analysis of DEGs specifically expressed at 3 d **(C)**, 9 d **(D)**, and 18 d **(E)**, respectively. **(F)** KEGG enrichment analysis of the common DEGs shared across all three waterlogging stages. The top 25 significantly enriched pathways are shown based on the rich factor and *P*-value.

We found 25,283 (10866 up- and 14417 down-regulated), 24615 (12019 up- and 12596 down-regulated), and 20236 (9498 up- and 10738 down-regulated) differentially expressed genes (DEGs) at 3 d, 9 d, and 18 d of treatment (false discovery rate [FDR]<0.05), respectively (**Figure 4A**; **Supplementary Figure 3**; **Supplementary Table 1**). Venn analysis revealed that 9795 DEGs were consistently expressed across all three time points (**Figure 4B**; **Supplementary Table 1**). This indicates that these genes are the core response factors of *H. hamabo* in response to waterlogging stress.

### KEGG pathway enrichment analysis of stage-specific and common DEGs

Kyoto encyclopedia of genes and genomes (KEGG) enrichment analysis was conducted to explore the stage-specific and common biological functions of DEGs (**Figures 4C–F**). At 3d, specific DEGs were predominantly involved in photosynthesis and plant hormone signal transduction, reflecting a rapid adjustment of energy metabolism in response to initial stress (**Figure 4C**). By 9 d, the functional focus shifted to metabolic pathways, the biosynthesis of secondary metabolites, fatty acid metabolism, and zeatin and fatty biosynthesis, indicates that *H. hamabo* undergoes a vigorous metabolic reconfiguration at this stage. By regulating the physicochemical characteristics of the cell membrane through lipid metabolic pathways, the plants promote the production of defensive secondary metabolites to respond to waterlogging stress (**Figure 4D**). The stage-specific DEGs at 18 d were significantly enriched in pathways including ribosome, plant hormone signal transduction, and ubiquitin-mediated proteolysis (**Figure 4E**), suggesting the robust adjustment of protein turnover and hormonal signaling during the late stage of waterlogging stress. Notably, the common DEGs shared by all stages were significantly enriched in phenylpropanoid biosynthesis, biosynthesis of secondary metabolites, MAPK signaling pathway, and the plant hormone signal transduction (**Figure 4F**). This demonstrates that regulating cell wall biosynthesis, maintaining hormone balance, and activating stress resistance signal transduction are the most fundamental and crucial response mechanisms of *H. hamabo* in the waterlogging process.

### Weighted gene co-expression network analysis of the core genes for waterlogging response in *H. hamabo*

**Figure 5:**
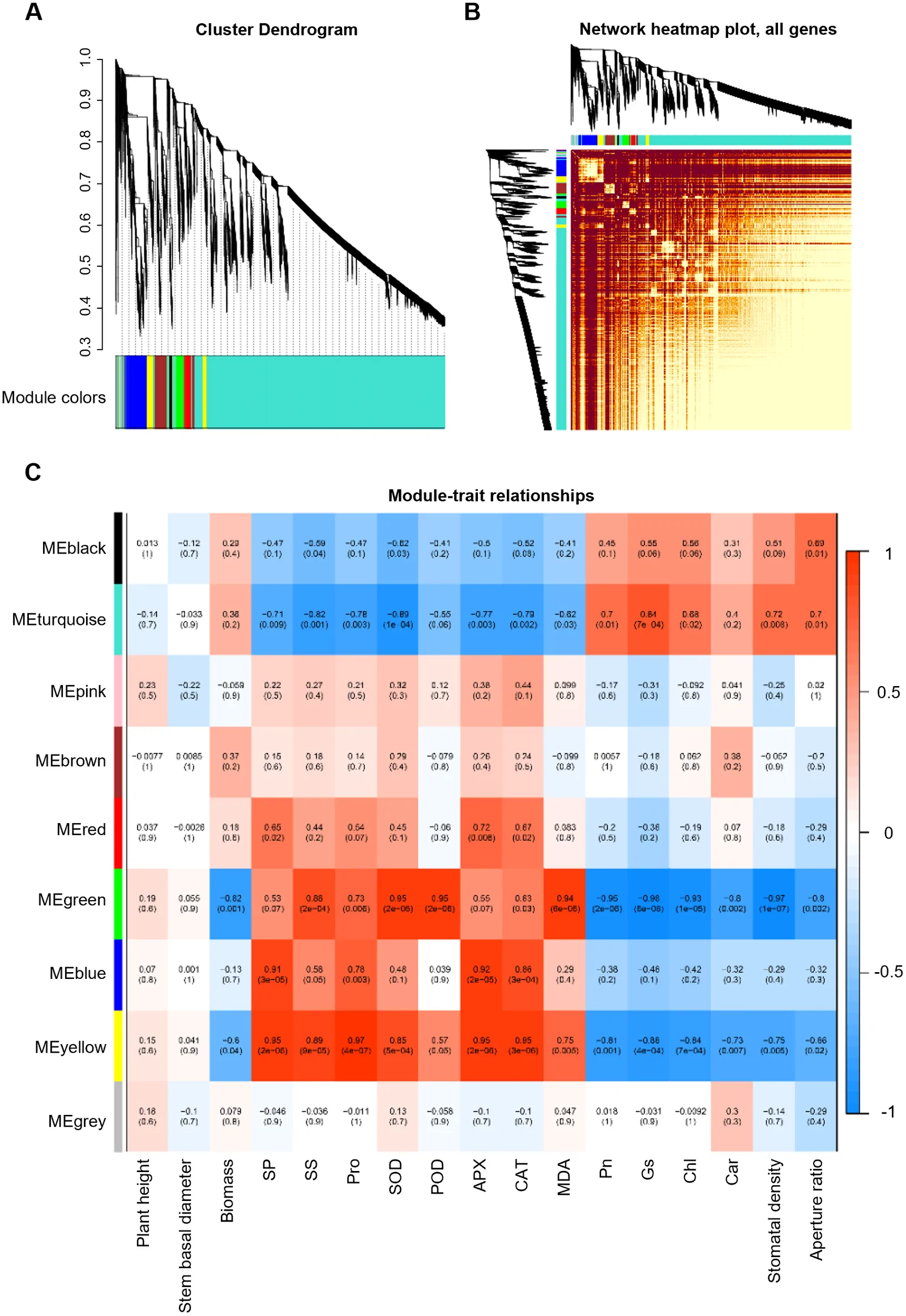
Weighted gene co-expression network analysis (WGCNA) in *H. hamabo* under waterlogging stress. **(A)** Hierarchical cluster dendrogram of the common differentially expressed genes (core DEGs). The branches represent individual genes, and the colors below indicate the assigned co-expression modules. **(B)** Heatmap of the topological overlap matrix (TOM) among all genes. Darker red indicates higher topological overlap. The main diagonal (TOM = 1 for each gene with itself) was set to NA prior to plotting to avoid visual saturation and to facilitate comparison of within- and between-module connectivity. **(C)** Heatmap of module-trait relationships. Rows correspond to distinct gene co-expression modules, and columns correspond to various physiological and biochemical traits. The numbers within each cell represent the Pearson correlation coefficient (top) and the corresponding *P*-value (bottom, in parentheses). The color scale on the right indicates the correlation level, with red representing positive correlations and blue representing negative correlations. SP, soluble protein; SS, soluble sugar; Pro, proline; SOD, superoxide dismutase; POD, peroxidase; APX, ascorbate peroxidase; CAT, catalase; MDA, malondialdehyde; Pn, net photosynthetic rate; Gs, stomatal conductance; Chl, total chlorophyll; Car, carotenoids.

We next performed weighted gene co-expression network analysis (WGCNA) on the common DEGs (core DEGs) identified at 3d, 9d, and 18d of waterlogging to investigate the persistent core regulatory network of *H. hamabo* in response to waterlogging stress. We conducted hierarchical clustering on the core DEGs (**Figure 5A**), and the topological overlap heatmap confirmed the reliability of the clustering, with genes within the modules exhibiting high expression synergy (**Figure 5B**). These core genes were clustered into 8 co-expression modules, marked with different colors.

To clarify the physiological functions driven by these modules, we performed Pearson correlation analysis between the 8 modules and different physiological and biochemical traits (**Figure 5C**). The results revealed that the transcriptional response of *H. hamabo* to waterlogging is primarily partitioned into two core regulatory camps: “defense activation” and “photosynthesis related module”.

For the defense camp, the MEyellow module was significantly and positively correlated with plant osmotic regulation, antioxidant defense, and the membrane lipid peroxidation (**Figure 5C**). Similarly, the MEgreen module exhibited highly positive correlations with SOD, POD, and MDA, indicating that the genes in these two modules constitute the core, continuously activated network responsible for stress resistance and damage response. Furthermore, the MEblue module demonstrated an extremely high positive correlation with APX and CAT, suggesting the existence of a highly specific sub-network dedicated to targeted ROS scavenging alongside the defense system.

Conversely, the MEturquoise module, we designate as the photosynthesis related module, showed a strong positive correlation with photosynthetic parameters including stomatal conductance and chlorophyll content (**Figure 5C**). This positive correlation indicates that its expression is high under control conditions but continuously suppressed under waterlogging stress. Accordingly, this module is enriched with key genes involved in basic metabolism and photosynthetic that are actively downregulated during prolonged stress.

### Interaction network analysis and hub gene identification of core waterlogging-responsive genes in *H. hamabo*

We constructed co-expression interaction networks for four core functional modules (MEturquoise, MEgreen, MEblue, and MEyellow) identified via WGCNA, which were highly associated with stress defense and growth maintenance (Supplementary **Figure 4**), to further explore the core regulatory factors underlying the sustained waterlogging tolerance response of *H. hamabo* (**Supplementary Figure 4**). Within these networks, large nodes located in the central layers and exhibiting extremely high connectivity were defined as hub genes of their respective modules, typically serving as key “master switches” in transcriptional regulatory networks.

The growth and photosynthesis maintenance network (MEturquoise module) represents a fundamental metabolic pathway that is continuously suppressed under waterlogging stress. This module comprises 146 structural genes and 4 transcription factors (TFs). Furthermore, we identified hub genes such as TRINITY_DN15434_c0_g2 and TRINITY_DN150352_c0_g1 (**Supplementary Figure 4A**; **Supplementary Table 3**), which are likely key target factors regulating stomatal conductance, chlorophyll synthesis, and the allocation of photosynthetic energy flow under stress conditions.

The core defense and damage response networks (MEgreen and MEyellow modules; **Supplementary Figures 4B, C**) exhibit a highly dense and complex regulatory interaction network. In the MEgreen module, we identified 137 structural genes and 12 TFs, among which genes such as TRINITY_DN1575_c0_g2 and TRINITY_DN9647_c1_g1 occupy the most central positions within the network (**Supplementary Figure 4B**; **Supplementary Table 4**). In the MEyellow module, 138 structural genes and 12 TFs were identified, among which 20 genes, including TRINITY_DN13065_c0_g1 and TRINITY_DN3874_c0_g1, serve as hub genes (**Supplementary Figure 4C**; **Supplementary Table 5**). These hub genes are highly likely to serve as the primary transcriptional regulators that comprehensively activate osmotic adjustment (e.g., massive proline synthesis) and the antioxidant defense system (e.g., SOD, POD), thereby determining the basal waterlogging tolerance ceiling of *H. hamabo*.

In addition, the MEblue module, which exhibits a specific and high correlation with the enzymatic activities of APX and CAT, comprises 77 structural genes and 12 transcription factors, with a few genes, such as TRINITY_DN22836_c0_g1 and TRINITY_DN3556_c0_g4, serving as hubs (**Supplementary Figure 4D**; **Supplementary Table 6**). This suggests that these core nodes may act as upstream elements that specifically target and regulate the expression of key antioxidant enzyme genes, mediating the fine-tuned reactive oxygen species (ROS) scavenging and adjustment mechanisms during the late stage of waterlogging.

In summary, through the analysis of gene interaction networks, we have successfully screened out key members of each core function from among hundreds of differentially expressed genes. These hub genes, characterized by extremely high interaction weights, provide highly precise candidate targets for future studies aimed at elucidating the molecular mechanisms underlying waterlogging tolerance in *H*. *hamabo* and for genetic engineering efforts toward stress tolerance improvement.

## Discussion

### Limited waterlogging tolerance and a conservative survival strategy in *H. hamabo*

*H. hamabo* is a semi-mangrove woody species that naturally occurs in coastal tidal flats, sandy beaches, and estuarine wetlands. As a dominant shrub in coastal shelterbelts, it frequently suffers from seasonal waterlogging and typhoon-induced storm surges in eastern China, resulting in significant growth inhibition and ecological stress (Li et al., 2012; Wang et al., 2019; Sakhanokho et al., 2020; Wang et al., 2022b). Our study demonstrates that *H. hamabo* exhibits a limited tolerance to waterlogging, with an average lethal time of approximately 18 days. This duration is shorter than the 40–100 days reported for other wetland species (Mommer et al., 2006; Liu et al., 2024), yet longer than the median lethal time of about 10 days for lotus (*Nelumbo nucifera*) (Deng et al., 2022), and comparable to the 7–16 days documented for rice (*Oryza sativa*) (Xu et al., 2006). Under waterlogging stress, *H. hamabo* exhibits a progressive decline in growth and photosynthetic capacity rather than sudden, catastrophic failure (**FigureS 1**, **2**; **Supplementary Figure 1**). This adaptive pattern contrasts sharply with *Nelumbo nucifera*, which is highly sensitive to waterlogging stress (Deng et al., 2022), and also diverges from the rapid “escape” elongation strategy employed by deepwater rice and certain wetland herbs (Hattori et al., 2009; Voesenek and Bailey‐Serres, 2015). Instead, *H. hamabo* employs a conservative “quiescence” strategy characterized by restrained growth, stabilized osmotic adjustment, and sustained antioxidant defense. Given its unique coastal semi-mangrove habitat, such a gradual and coordinated response represents an evolutionarily adaptive syndrome that balances survival and stress tolerance under frequent and fluctuating waterlogging regimes (Ni et al., 2021; Wang et al., 2022b). Unraveling these mechanisms not only deepens our understanding of flood adaptation in coastal woody plants but also supplies valuable gene resources for future breeding and shoreline ecological restoration.

### The influence of waterlogging on morphology and biomass

Prolonged waterlogging progressively suppressed plant height, ground diameter growth, and biomass accumulation in *H. hamabo*, with inhibitory effects intensifying as stress duration increased (**Figure 1**). Concurrently, typical phenotypic injuries occurred, including leaf chlorosis and abscission. These growth restrictions are closely associated with hypoxia-induced energy deficits and photosynthetic decline, which are widely recognized as core physiological constraints under waterlogging stress (Deng et al., 2022; Wang et al., 2022b). Hypoxia limits aerobic respiration and ATP production, while impaired photosynthesis further reduces carbon assimilation, leading to insufficient energy supply for cell elongation and division (Kreuzwieser and Rennenberg, 2014; Voesenek and Bailey‐Serres, 2015; Deng et al., 2022). Such growth inhibition was not merely a passive injury, but an active adaptive strategy employed by *H. hamabo* to conserve limited energy and prioritize survival under prolonged flooding (Bailey-Serres and Voesenek, 2008; Deng et al., 2022). Similar growth-inhibition strategies have also been reported in lotus (*Nelumbo nucifera*) and the mangrove plant *Kandelia obovata* (Deng et al., 2022; Liu et al., 2024). In *Nelumbo nucifera*, prolonged submergence suppresses leaf expansion and biomass accumulation to maintain energy balance (Deng et al., 2022). In *Kandelia obovata*, waterlogging significantly inhibits growth, development, and biomass production, with the degree of inhibition positively correlated with the duration of waterlogging stress (Liu et al., 2024). The stability in adversity demonstrated by *H. hamabo* may be attributed to its unique semi-mangrove adaptive characteristics in saline-waterlogged coastal wetlands (Colmer and Flowers, 2008; Wang et al., 2019; Ni et al., 2021; Wang et al., 2022b; Mao et al., 2023). These results indicate that the trade-off between growth inhibition and energy conservation represents a conserved and crucial survival mechanism in coastal woody plants facing frequent waterlogging events.

### Effects of waterlogging on photosynthetic system and stomatal traits

Waterlogging caused a significant reduction in chlorophyll content in *H. hamabo*, indicating accelerated chlorophyll degradation under hypoxia stress (**Figure 2**). The loss of chlorophyll directly weakened light-harvesting capacity and reduced the efficiency of light absorption, thereby limiting the light reaction process of photosynthesis (Kreuzwieser and Rennenberg, 2014; Sharma et al., 2022; Wang et al., 2022b; Eerdekens et al., 2025; Zhang et al., 2025). Meanwhile, waterlogging induced marked stomatal closure and reduced stomatal density, which restricted CO_2_ diffusion into intercellular spaces and thus led to a sharp decline in net photosynthetic rate (Pn) (**Figures 2C–G**). Such non-stomatal and stomatal limitations synergistically contributed to the rapid suppression of photosynthesis under waterlogging, as widely reported in woody wetland plants (Li et al., 2011; Sharma et al., 2022; Mao et al., 2023; Saha and Johnson, 2025; Zhang et al., 2025). In addition, carotenoid content also decreased under prolonged waterlogging (**Figure 2B**), weakening the non-photochemical quenching and photoprotective capacity of leaves and further aggravating photooxidative damage to the photosynthetic apparatus (Kreuzwieser and Rennenberg, 2014; Eerdekens et al., 2025; Zhang et al., 2025). Transcriptomic analysis further confirmed that genes involved in photosynthesis, light harvesting, and chlorophyll metabolism were significantly changed during the early stage of waterlogging (**Figure 4**). Taken together, the coordinated downregulation of photosynthetic structure, pigment synthesis, and stomatal function constitutes a core strategy of *H. hamabo* in response to waterlogging-induced energy crisis and oxidative stress.

Transcriptomic data further corroborated these physiological phenotypes. KEGG enrichment analysis revealed that DEGs at day 3 of waterlogging were markedly enriched in photosynthesis-associated pathways (**Figure 4C**), indicating that rapid transcriptional reprogramming of the photosynthetic machinery occurs during the early stage of hypoxia. Furthermore, the MEturquoise module, a photosynthesis-associated module, exhibited significant positive correlations with net photosynthetic rate (Pn), stomatal conductance (Gs), and chlorophyll content (**Figure 5C**). Consistently, its expression was markedly suppressed throughout the entire waterlogging period. This module, containing 146 structural genes and 4 transcription factors, likely represents a core transcriptional network that regulates photosynthetic maintenance; its downregulation is the fundamental cause of the gradual decline in photosynthetic capacity. The coordinated downregulation of photosynthetic genes at the transcriptomic level, together with the decline in corresponding physiological parameters, collectively constitutes compelling evidence for hypoxia-driven photosynthetic shutdown. This global pattern of photosynthetic gene suppression has been similarly documented in mulberry (*Morus alba* L.): Waterlogging treatment significantly downregulated genes encoding LHCII proteins, LHCI antenna proteins, the oxygen-evolving complex, cytochrome b6/f, and calvin cycle enzymes, leading to a concurrent decline in Pn, Gs, and chlorophyll content (Su et al., 2022). The consistency of these findings suggests that transcriptional repression of the photosynthetic apparatus is a prevalent and conserved response mechanism to hypoxic stress in woody plants, although the specific hub genes involved may vary among species.

### Dynamic responses of osmotic adjustment and antioxidant systems to waterlogging stress

Prolonged waterlogging triggered pronounced dynamic adjustments in osmolyte accumulation and antioxidant enzyme activities in *H. hamabo* (**Figure 3**). The contents of proline and soluble proteins all increased initially and then declined under prolonged stress (**Figures 3B, C**), indicating an active osmotic adjustment strategy to maintain cell turgor and stabilize macromolecular structures at the early stage, whereas prolonged hypoxia disrupted osmolyte synthesis and cellular homeostasis (Mao et al., 2023; Zhang et al., 2025). For antioxidant enzymes, SOD and POD activities continuously increased throughout the stress period, serving as the first-line defense to scavenge superoxide anions and hydrogen peroxide (**Figures 3D, E**). In contrast, APX and CAT activities peaked at the middle stage and then decreased slightly (**Figures 3F, G**), suggesting that the ascorbate-glutathione cycle and peroxisomal ROS scavenging were gradually constrained under prolonged hypoxia (Kreuzwieser and Rennenberg, 2014; Eerdekens et al., 2025). Concurrently, MDA content continuously increased with stress duration (**Figure 3H**), demonstrating that membrane lipid peroxidation was aggravated over time and oxidative damage could not be fully avoided despite activated antioxidant defense. Notably, similar physiological patterns were observed in the mangrove species *Kandelia obovata* under prolonged flooding stress. With increasing inundation duration, the contents of MDA, SOD, GSH, soluble sugar, soluble protein, and proline decreased, whereas POD and APX activities increased significantly, indicating a conserved antioxidant strategy in flood‑adapted coastal woody species (Liu et al., 2023b). Therefore, the activated antioxidant system effectively alleviated oxidative injury at the early and middle stages of waterlogging, but failed to reverse progressive cellular damage under long-term stress, which is consistent with adaptive patterns reported in coastal woody plants (Wang et al., 2022b; Mao et al., 2023).

WGCNA-based co-expression network analysis clarified the correspondence between physiological responses and functional gene modules. The MEyellow module exhibited significant positive correlations with osmotic adjustment parameters (proline, soluble sugars, soluble proteins), antioxidant enzyme activities (SOD, POD), and MDA content (**Figure 5C**). This association pattern suggests that the MEyellow module serves as a core hub integrating the functions of osmotic adjustment, reactive oxygen species scavenging, and membrane damage sensing. Within this module, 20 hub genes, including TRINITY_DN13065_c0_g1 and TRINITY_DN3874_c0_g1, exhibited high network connectivity (**Supplementary Figure 4C**), These may function as master regulators orchestrating multidimensional defense responses. Similarly, the MEgreen module was highly correlated with SOD, POD, and MDA (**Figure 5C**), with hub genes such as TRINITY_DN1575_c0_g2 and TRINITY_DN9647_c1_g1 positioned at the center of the network (**Supplementary Figure 4B**). Notably, the MEblue module exhibited a specific positive correlation with APX and CAT (**Figure 5C**), but not with SOD or POD, suggesting the existence of a dedicated H_2_O_2_-scavenging subnetwork that functions in concert with the overall antioxidant defense system. This modular specialization is consistent with the distinct temporal patterns of these enzymes: SOD and POD activities increased continuously throughout the entire 18-day stress period, whereas APX and CAT activities peaked on day 9 and then gradually declined (**Figures 3D–G**). The specific association of the MEblue module with APX and CAT, rather than SOD or POD, indicates that distinct transcriptional modules separately regulate different branches of the ROS scavenging system, thereby enabling precise temporal control. This pattern in antioxidant defense has also been observed in *Kandelia obovata*: long-term waterlogging stress can induce differential changes in various antioxidant enzymes (Liu et al., 2023b). In *Taxodium mucronatum*, WGCNA identified independent functional modules related to the activity of specific antioxidant enzymes (Gao et al., 2025).

### Stage-specific transcriptomic responses to waterlogging

Transcriptomic profiling revealed distinct stage-specific molecular strategies employed by *H. hamabo* in adaptation to progressive waterlogging stress. At the early stage (3d), differentially expressed genes (DEGs) were predominantly enriched in photosynthesis and hormone signal transduction (**Figure 4C**), indicating rapid perception and primary metabolic reprogramming to cope with sudden hypoxia and reduced carbon fixation (Wang et al., 2022b; Zhang et al., 2025). This early response reflects an urgent shift to stabilize energy supply and restrain redundant growth. At the middle stage (9d), the transcriptome shifted markedly toward secondary metabolism, zeatin and fatty biosynthesis (**Figure 4D**), suggesting enhanced stress signal transmission and activation of defense capabilities to alleviate oxidative damage under persistent hypoxia (Liu et al., 2023b; Mao et al., 2023). This stage represents a strengthened defensive phenotype to resist submergence-induced injuries. At the late stage (18d), the dominant transcriptional programs switched to ribosomes, plant hormone signal transduction, and ubiquitin-mediated protein degradation pathways (**Figure 4E**), indicating that *H. hamabo* initiates protein turnover, damaged protein clearance, hormone rebalancing, and translational regulation to sustain cellular homeostasis and promote stress adaptation and recovery under prolonged waterlogging (Wang et al., 2022b; Zhang et al., 2025).

Secondary metabolites are essential for plant survival, mediate key processes underlying environmental adaptation, and are closely associated with indirect defense mechanisms (Kutchan, 2001; Richter et al., 2015; Alseekh and Fernie, 2018; Liu et al., 2024). As multifunctional bioactive compounds, they participate extensively in diverse stress responses, including salinity, heavy metal toxicity, and other abiotic challenges (Kumar et al., 2012; Lushchak and Semchuk, 2012; Wang et al., 2016; Li et al., 2019; Jayaraman et al., 2021; Liu et al., 2024). In this study, the commonly shared differentially expressed genes (DEGs) across all waterlogging stages were significantly enriched in phenylpropanoid biosynthesis, and biosynthesis of secondary metabolites (**Figure 4F**). These results demonstrate that modulation of cell wall biosynthesis, maintenance of hormonal homeostasis, and activation of stress-resistant signal transduction represent the most fundamental and conserved adaptive mechanisms employed by *H. hamabo* throughout the waterlogging process. In particular, the sustained upregulation of the phenylpropanoid pathway contributes to enhanced lignin deposition and secondary cell wall thickening, which strengthen cell mechanical stability, reinforce root and leaf structural integrity, and alleviate hypoxia-induced oxidative damage under long-term waterlogging stress (Ranathunge et al., 2011; Shiono et al., 2022).

Similar results have also been observed in other species. In wheat, waterlogging treatment significantly reduced stem lignin content and transcriptionally suppressed key lignin biosynthesis genes, including phenylalanine ammonia-lyase 6 (PAL6), cinnamoyl-CoA reductase 2 (CCR2), and ferulate 5-hydroxylase 2 (F5H2), indicating that lignin metabolism is a primary target of hypoxia-induced transcriptional reprogramming (Nguyen et al., 2016). In contrast, in barley, waterlogging-tolerant genotypes maintained higher expression levels of peroxidase (PER) and cinnamoyl-CoA reductase (CCR) genes in the phenylpropanoid biosynthesis pathway compared to susceptible genotypes, which contributed to enhanced cell wall integrity and improved waterlogging tolerance (Wang et al., 2024). These results suggest that activation of phenylpropanoid biosynthesis is a functionally conserved yet quantitatively variable adaptive strategy; the degree of its transcriptional maintenance may determine the upper limit of plant waterlogging tolerance.

## Data Availability

The data presented in the study are deposited in the National Center for Biotechnology Information Sequence Read Archive repository, accession number PRJNA1467080.
