# Supplementary material for: Integrated morpho-physiological and transcriptomic dissection of waterlogging tolerance in Hibiscus hamabo
Source: Front Plant Sci. 2026 Sep 11;17:1882944. doi: 10.3389/fpls.2026.1882944 (PMC13612267; doi:10.3389/fpls.2026.1882944)
Supplement: Supplementary file 1 [file DataSheet1.pdf]

Supplementary Figure 1

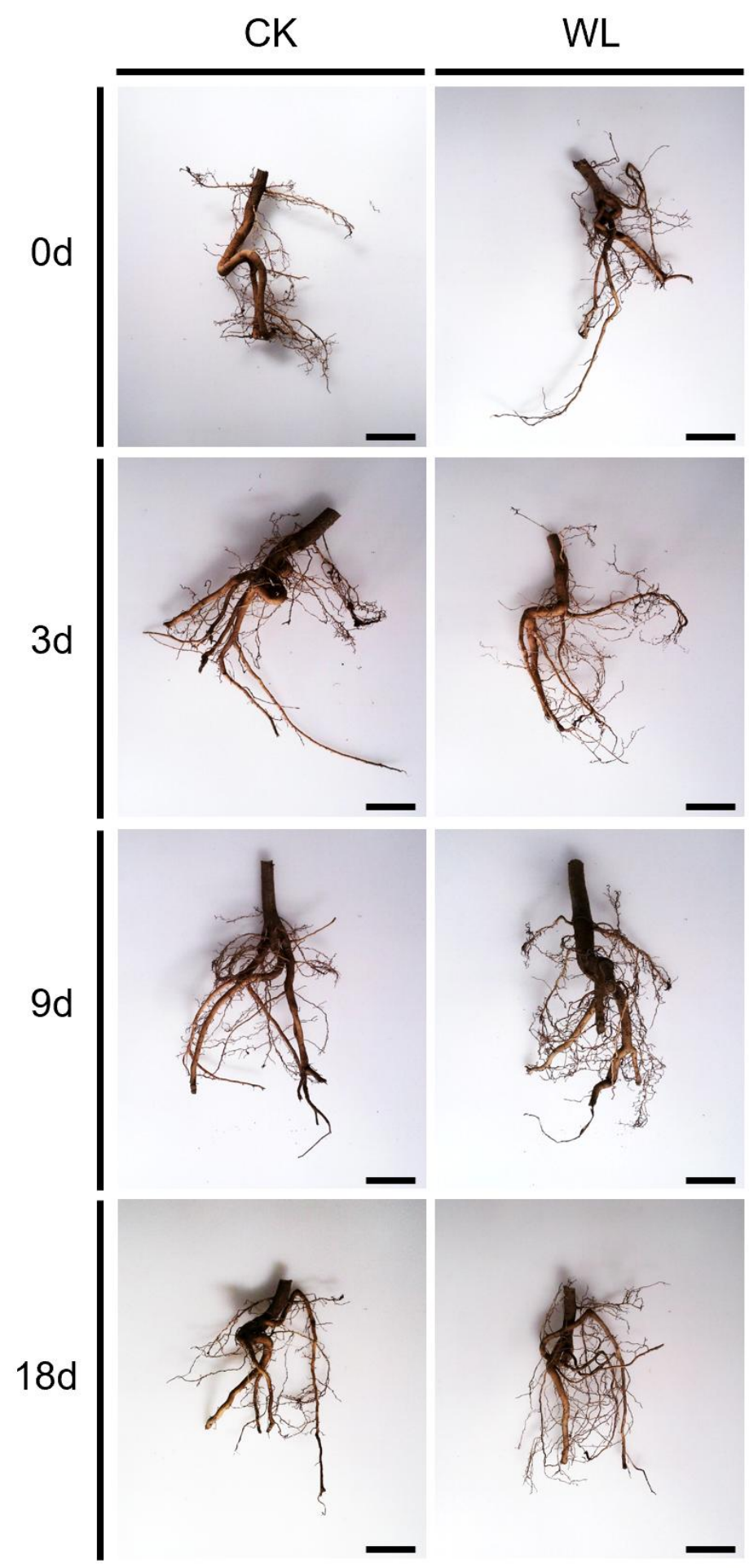

Supplementary Figure 1. The effects of waterlogging stress on the root growth of *H. hamabo*. Phenotypes of one-year-old *H. hamabo* under waterlogging stress (WL) and non- waterlogging (CK) conditions at 0, 3, 9, and 18 days of treatment. Scale bars = 3 cm.

Supplementary Figure 2

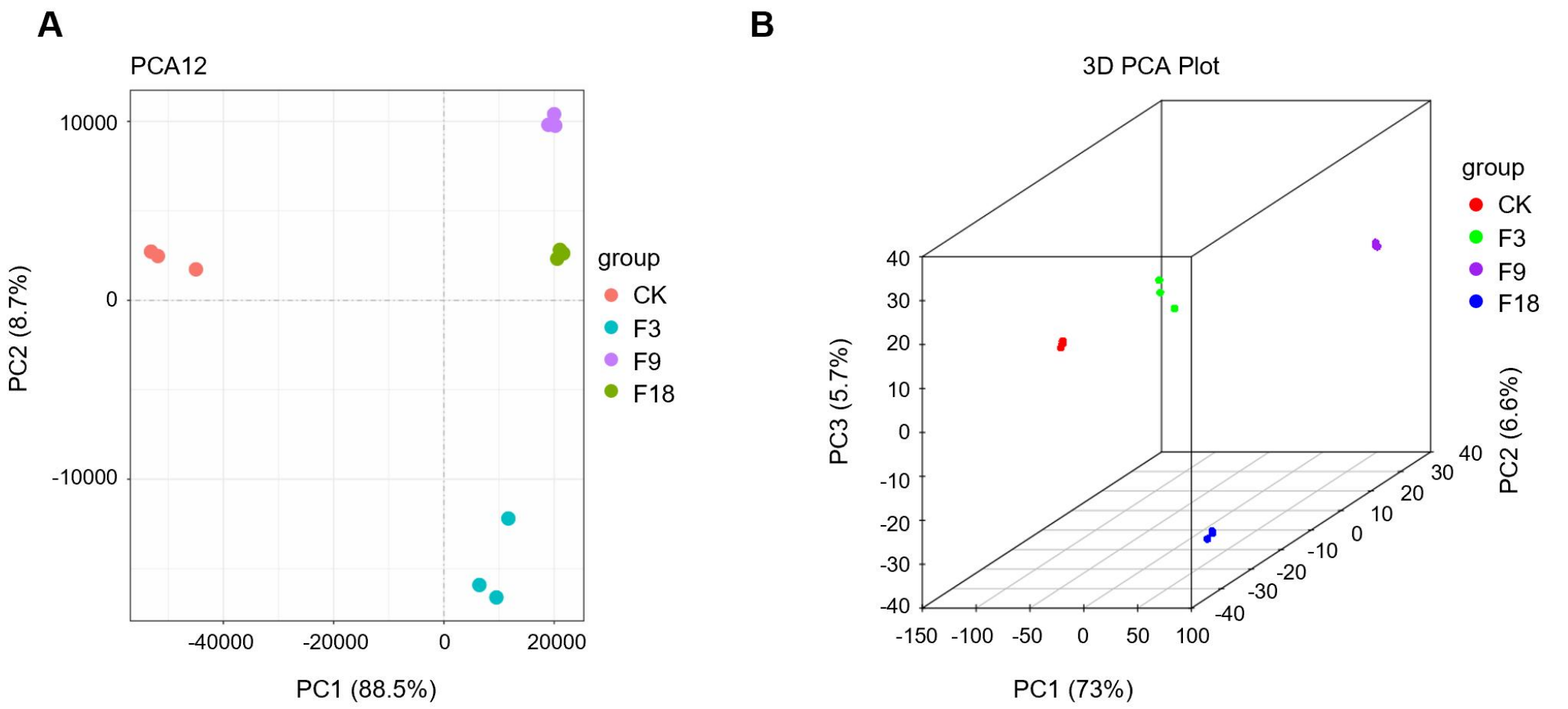

Supplementary Figure 2. Principal component analysis (PCA) of the transcriptomic profiles in *H. hamabo* under waterlogging stress. (**A**) Two-dimensional (2D) PCA plot based on global gene expression levels. The x-axis and y-axis represent principal component 1 (PC1) and principal component 2 (PC2), accounting for 88.5% and 8.7% of the total variance, respectively. (**B**) Three-dimensional (3D) PCA plot illustrating the spatial distribution of samples across the first three principal components: PC1 (73%), PC2 (6.6%), and PC3 (5.7%). Each dot represents an individual biological replicate. Different colors denote distinct experimental groups: CK (control), F3 (waterlogging for 3 d), F9 (waterlogging for 9 d), and F18 (waterlogging for 18 d). The tight clustering of biological replicates within each group indicates high reproducibility of the

Supplementary Figure 3

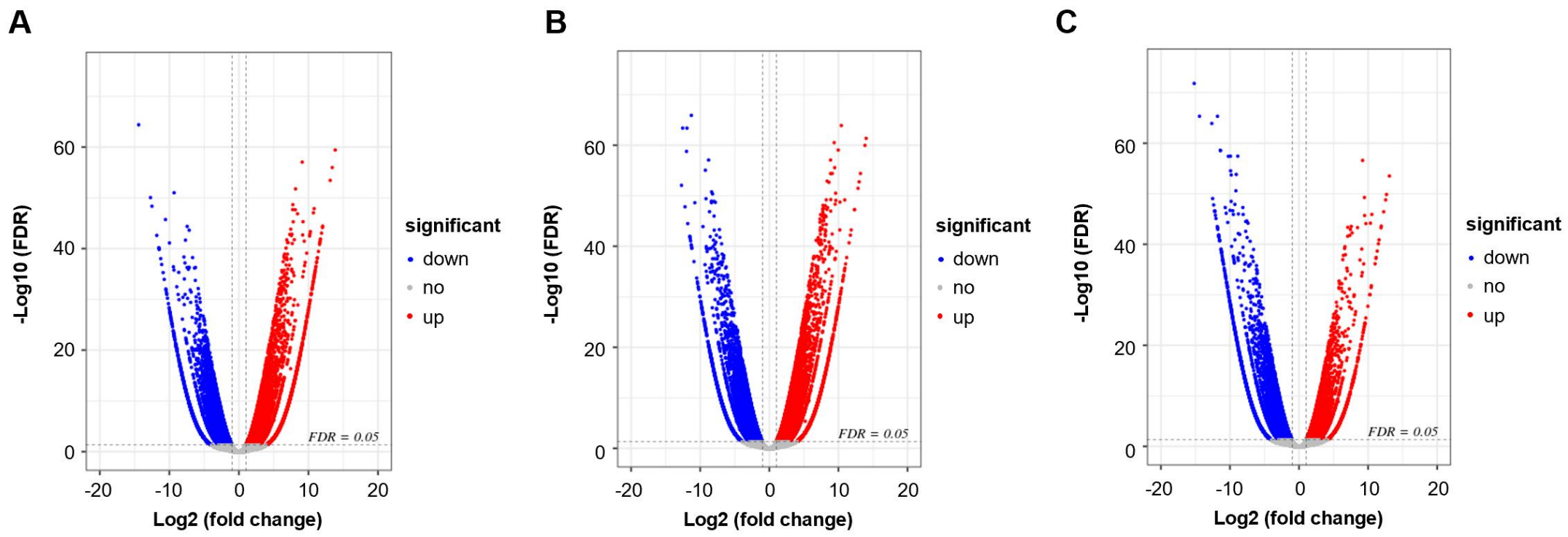

Supplementary Figure 3. Volcano plots of differentially expressed genes (DEGs) in *H. hamabo* under waterlogging stress. (**A-C**) Volcano plots displaying the distribution of DEGs between the waterlogged (WL) and control (CK) plants at 3 d (**A**), 9 d (**B**), and 18 d (**C**) of treatment, respectively. The x-axis represents the magnitude of gene expression changes expressed as Log2(fold change), and the y-axis represents the statistical significance expressed as -Log10(FDR). Each dot represents an individual gene. Red dots indicate significantly up-regulated genes, blue dots indicate significantly down-regulated genes, and grey dots represent genes with no significant differential expression. The horizontal dotted line indicates the significance threshold of false discovery rate (FDR) = 0.05.

Supplementary Figure 4

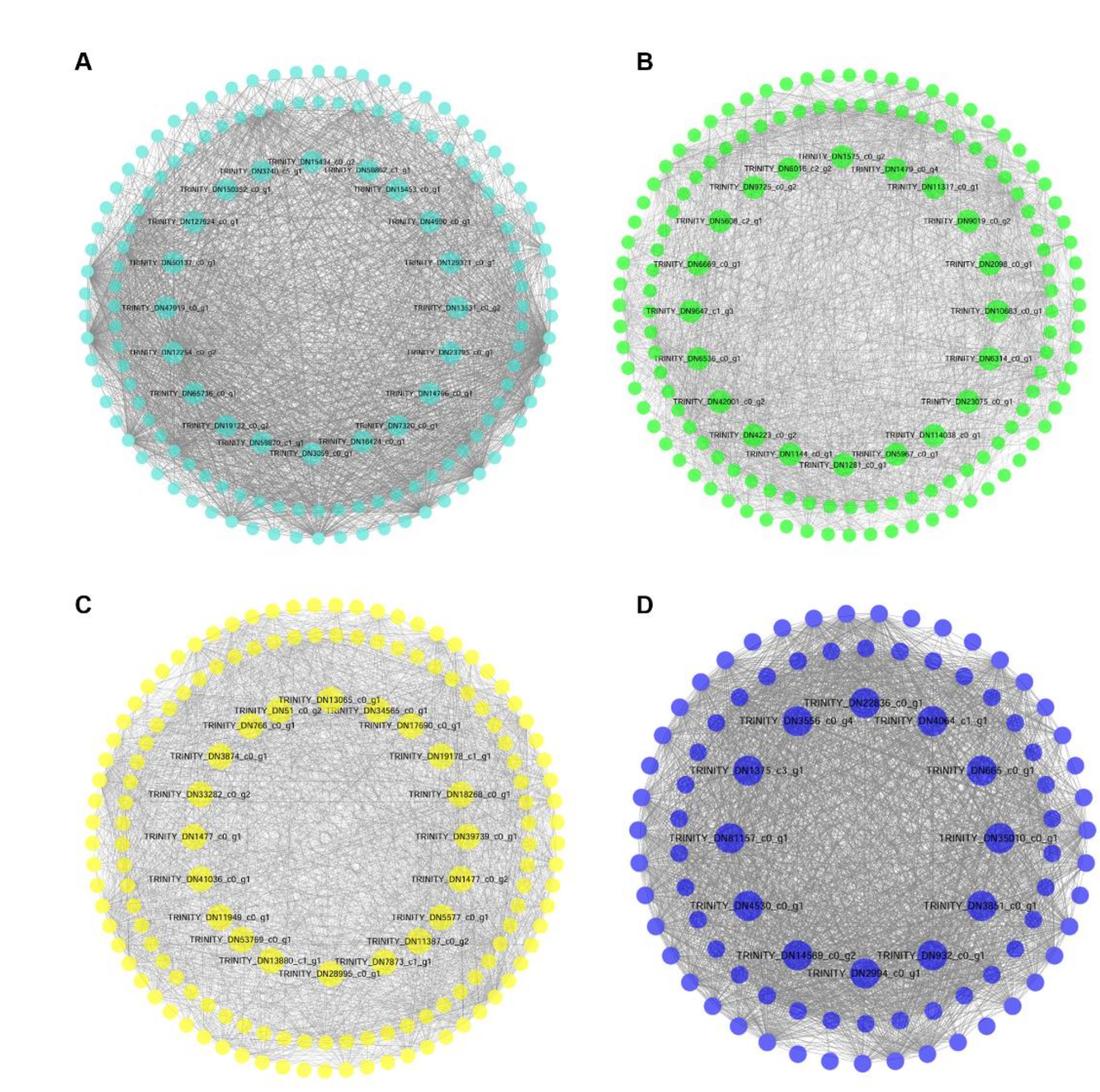

Supplementary Figure 4. Interaction network analysis of core functional modules of *Hibiscus hamabo* under waterlogging stress. (**A-D**) Interaction networks of genes within the MEturquoise (**A**), MEgreen (**B**), MEyellow (**C**), and MEblue (**D**) modules, respectively. Each circular node represents an individual differentially expressed gene, and the connecting lines indicate topological overlap and co-expression relationships between genes. The size of each node is proportional to its network connectivity (k-within degree); larger nodes located in the center of the networks represent the key hub genes with the highest regulatory weights.
